# Preliminary Effect and Acceptability of an Intervention to Improve End-of-Life Care in Long-Term-Care Facilities: A Feasibility Study

**DOI:** 10.3390/healthcare9091194

**Published:** 2021-09-10

**Authors:** Chihiro Yamagata, Sachiko Matsumoto, Mitsunori Miyashita, Yusuke Kanno, Atsuko Taguchi, Kana Sato, Hiroki Fukahori

**Affiliations:** 1 Graduate School of Health Care Sciences, Tokyo Medical and Dental University (TMDU), Tokyo 113-8510, Japan; satok.kanr@tmd.ac.jp; 2 School of Nursing, Tokyo Women’s Medical University, Tokyo 162-8666, Japan; 3 Seirei Fujisawa Welfare Town, Kanagawa 251-0861, Japan; s.matsumoto@sis.seirei.or.jp; 4 Department of Palliative Nursing, Health Sciences, Tohoku University Graduate School of Medicine, Sendai 980-8575, Japan; miya@med.tohoku.ac.jp; 5 Nursing Course, School of Medicine, Yokohama City University, Kanagawa 236-0004, Japan; ykanno@yokohama-cu.ac.jp; 6 Faculty of Nursing and Medical Care, Keio University, Kanagawa 252-0883, Japan; ataguchi@sfc.keio.ac.jp (A.T.); fukahori@sfc.keio.ac.jp (H.F.)

**Keywords:** allied health personnel, long-term care, nurses, residential facilities

## Abstract

The number of deaths of older adults in long-term care settings will increase with the aging population. Nurses and care workers in these settings face various challenges in providing end-of-life care, and interventions for quality end-of-life care may be useful. This feasibility study aims to explore the preliminary effect and acceptability of an intervention named the EOL Care Tool to improve end-of-life care in long-term-care facilities. We conducted a single-arm quasi-experimental study using mixed methods. This tool consisted of multiple components: professionalized lectures, newly developed structured documents, regular conferences regarding end-of-life care, and educational support from administrators. Twenty-four nurses and fifty-five care workers employed in a long-term care facility participated. For nurses, improvement in attitudes toward end-of-life care (*p* < 0.05) and interdisciplinary collaboration (*p* < 0.05) were shown quantitatively. Regarding acceptability, nurses and care workers evaluated the tool positively except for the difficulty of using the new documents. However, qualitative results showed that care workers felt the reluctance to address the work regarding end-of-life care. Therefore, a good preliminary effect and acceptability for nurses were indicated, while acceptability for care workers was only moderate. Revision to address the mentioned issues and evaluation of the revised tool with a more robust research design are required.

## 1. Introduction

The older population has grown globally in recent years [1], and it is estimated that more people will die in long-term care (LTC) settings in the future [2]. In Japan, one of the most aged societies in the world, the national government promotes end-of-life care for older adults in LTC settings as well as a community [3], because about 72% of deaths occur in hospitals [4] though many Japanese people want to avoid death in the hospital in national surveys [5]. The number of people dying in LTC settings in Japan has increased by 9.2% in the last twenty years [4], and it is essential to maintain and improve the quality of end-of-life care in these settings. In LTC settings, care workers have more opportunities to be involved in caring for residents than nurses, though care workers’ medical and end-of-life care education is often insufficient [6,7,8]. In addition, as the involvement of healthcare professionals in care for residents is relatively low, end-of-life care in LTC settings addressing the changes in physical and mental symptoms of residents can be limited. Therefore, in countries such as Japan, where the population is rapidly aging, there is an urgent need to improve the quality of end-of-life care in the LTC setting.

Nurses and care workers in LTC settings face various challenges in providing end-of-life care. Nurses in LTC settings have critical roles in maintaining quality end-of-life including coordinating care during an uncertain prognosis, mediating residents’ wishes in end-of-life care decisions, managing symptoms, and supporting relatives of older residents [9] who often have multiple chronic diseases with unstable conditions and symptoms [10,11]. Care workers also involve end-of-life care for residents with unstable physical and psychological status, provide daily direct care to residents [12], and support residents in their IADL and ADLs until the end of life. In addition, nurses and care workers in LTC facilities are required to cope with the preferences of residents and their families, because LTC facilities are where residents spend years in the last stage of their lives [13,14]. Their careful observation of symptoms and building of relationships with older residents and their families is necessary for quality end-of-life care. However, both nurses and care workers experience difficulty and lack of confidence in addressing care around death and dying in LTC settings [15,16]. Due to different educational backgrounds between nurses and care workers, information-sharing does not work well, and interdisciplinary collaboration is consequently difficult. Therefore, intervention to improve end-of-life care confidence and interdisciplinary collaboration between nurses and care workers is necessary to provide quality end-of-life care to the residents.

Regarding interventions to improve the quality of end-of-life care in LTC settings, various interventions have been developed in the West, including the program beyond the countries for improving palliative care in nursing homes (PACE program) [17], educational programs or interventions for improving end-of-life care and symptom management for dementia patients in nursing homes [18,19,20], and educational programs for end-of-life care in nursing homes [21]. Further, the End-of-Life Nursing Education Consortium Geriatric (ELNEC-G) [22], an educational program to improve palliative care and end-of-life care, has developed in the United States (US) and is contributing to the education for licensed nurses and nursing assistants. Among these interventions, integrated care pathways, which are interventions combining multiple components, have been examined mainly in Western countries [10,23,24,25,26]. The Liverpool Care Pathway, a major example of an integrated care pathway, was developed in the United Kingdom (UK) to provide hospice care in settings other than formal hospices [27]. Previous research suggests that these interventions with multiple components, such as staff education and structured documents, are useful in increasing staff confidence in end-of-life care, teamwork, and communication in residential care facilities [23,28].

However, several challenges must be considered in developing a multi-component intervention to improve end-of-life care in regions other than the West, including Asia. In the UK, inappropriate care owing to an insufficient assessment of the patient’s condition and inadequate communications with families have caused serious problems in the use of the Liverpool Care Pathway [29]. Therefore, this type of intervention should be carefully developed and used so that appropriate assessment and communication can be assured at the end-of-life stage. Focusing on communication with older residents and their families is especially important because preference regarding end-of-life care and beliefs about death and dying vary depending on country or region [30].

Recently, the Japanese national government has recommended that care workers, not only healthcare professionals, be involved in shared decision-making in end-of-life care for patient advocacy [31]. As a result, the involvement of nurses and care workers in end-of-life care in LTCFs is expected to increase in the future. However, education regarding end-of-life care is insufficient, especially for care workers [6,7,32], and interventions to improve end-of-life care in LTCFs are scant in Japan [33]. Additionally, care workers have high anxiety toward end-of-life care and feel it to be a burden [7,32]. Discussions pertaining to residents’ intention regarding end-of-life tend to be avoided in LTC facilities in Japan [34]. Therefore, new interventions for quality end-of-life care should be developed for nurses and care workers in LTC settings.

When developing interventions, it is necessary to explore their preliminary effects and the reaction of the target population (e.g., nurses and care workers in this study), to understand the effect/impact of the intervention for them and what modifications are needed. To explore whether the intervention can work (e.g., the preliminary effect) and participants’ reaction/perception of the involvement of the intervention (e.g., acceptability), a feasibility study is helpful [35]. The aim of this study was to explore the preliminary effect and acceptability of the intervention named the “End-of-life Care Tool” for nurses and care workers in LTC settings in Japan.

## 2. Method

### 2.1. Design

This study adopted a single-group, quasi-experimental design using mixed methods to enhance our understanding of the potential range of outcomes and the reaction to the intervention among participants and to deepen the interpretations and conclusions from the participants’ subjective experience [36].

### 2.2. Setting

The study took place in an LTC facility—a “fee-based home for the elderly” (*yuryo-rojin-homu* in Japanese) with a clinic. The facility has three sections depending on the state of residents: low support section, high support section, and a facility-attached clinic. The LTC facility supports and provides care for residents along a continuum of living environments according to the daily life and healthcare needs of residents. Residents can move between settings with different levels of care as needed. They often live in these LTC facilities for years, and many residents die there. Most care workers were in the low- and high-support sections, and most nurses were in the clinic.

The features of the LTC facility in this study were the following:

1. A low-support section (approximately 400 residents): Most residents here were living independently or with assistance. Nurses and care workers supported residents mainly in IADLs. Residents’ health was relatively stable.
2. A high-support section (approximately 40 residents): Similar to a nursing home, many residents here were provided care mainly in ADLs, were bedridden, and/or were approaching death.
3. A facility-attached clinic (approximately 20 residents): This accepts residents from the other two sections as outpatients or inpatients for medical treatment or advanced care. More residents were approaching death here than in the other two sections.

### 2.3. Participants

Participants in the survey and interviews were nurses and care workers working in this facility. Inclusion criteria were engagement in hands-on care for residents; ability to read, write, and communicate in Japanese; and participation in the EOL Care Tool implementation. For the interview, purposive sampling was used to obtain diverse perspectives [37]. We asked the managers of each section to include staff members with various perspectives toward the EOL Care Tool.

### 2.4. Intervention

#### 2.4.1. Development of the Intervention

We developed a new intervention based on our literature review and the expertise of researchers (presents authors) and clinical experts as follows. We conducted a literature review regarding interventions to improve end-of-life care in LTC facilities [38] and identified the key intervention components for improving the quality of end-of-life care: education, documents, regular conferences, and educational support. The researchers and clinical experts (researchers, clinical managers of nurses and care workers, and a certified nurse specialist (CNS), who was an advanced practice nurse with a master’s degree) had repeatedly discussed the content of the intervention and the acceptability of the tool. Consequently, the intervention was developed based on the identified components. (Table 1; UMIN Clinical Trial Registry: UMIN000022579).

#### 2.4.2. The EOL Care Tool Intervention

“End-of-life care lectures” for nurses and care workers were developed based on parts of the Japanese version of the End-of-Life Nursing Education Consortium Geriatric (ELNEC-JG) and other related literature. The ELNEC-JG is a structured educational program for end-of-life care based on the original version developed in the US [22]. Participants learned the following in the six module lectures: “what is end-of-life care, “cultural and ethical issues,” “communication, loss, grief, and bereavement,” “pain management,” “symptom management,” “care in dying phase and death,” and “how to use the structured documents” (Table 1). The lectures were provided both face-to-face and by DVD, helping ensure the participation of all participants.

“Structured documents shared by the interdisciplinary team” include items related to the residents’ preferences, thoughts, and content related to their physical and mental status. These documents were intended to record and share information among nurses and care workers and to be used in regular conferences. The documents included four types of sheets for assessment, conference, daily records, and preparation for imminent death. Conference sheets contained items that referred to literature related to frailty: changes in weight, activity, walking, depression, food intake, etc. [39]. Daily records included items of symptom management and a list of required care.

In “regular conferences on end-of-life care”, the structured documents promote staff to share residents’ information regarding end-of-life care. The conferences were conducted regularly to cope with residents’ physical and psychological changes, wishes, or anxieties about the end of life. Before every regular conference, nurses and care workers had to talk with residents and/or their families about the end of life using structured documents.

**Figure 1:**
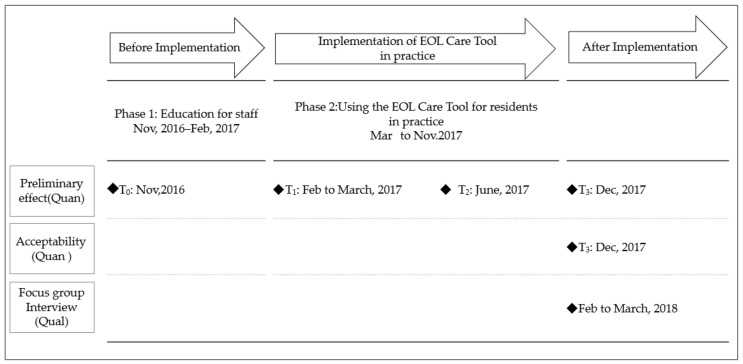
Overview of the design of the intervention and assessment. Ns: Nurse; Cw: Care worker; Quan: Quantitative approach; Qual: Quantitative approach.

As “educational support,” managers of both nurses and care workers provided advice and hands-on feedback on-site and in conferences. This support was provided because it was assumed that staff would have trouble discussing end-of-life care and may experience confusion due to the newly developed structured documents (Figure 1).

End-of-life care lectures were held for four months before launching the other three components; those three components were applied for nine months.

During the research periods, among 141 eligible residents, 134 residents (95.0%) were provided care by using the end-of-life care tool in the facility. This tool was not used for seven residents for the following reasons: the resident and/or their family did not want to use the tool; the resident was emotionally unstable, or death was imminent; some close relatives did not accept the resident’s approaching death, or the resident became hospitalized outside the facility. Among 134 residents, there were 73 (54.4%) in the low-support section, 44 (32.8%) in the high-support section, and 17 (12.7%) in the clinic. During the implementation of the tool, five residents in the clinic and three in the high-support section died.

### 2.5. Data Collection

Quantitative data were collected from November 2016 to December 2017; at four time-points: baseline (T_0_), after end-of-life care lectures (T_1_), and three and nine months after the EOL Care Tool implementation in residents’ care (T_2,_ T_3_; Figure 1). We performed data linkage using IDs attached to the questionnaires. Qualitative data were collected from February to March 2018.

#### 2.5.1. Characteristics of Participants

Characteristics of participants were collected including gender, age, occupation, educational level, years of experience in their profession, and years of working in the current workplace.

#### 2.5.2. Quantitative Data

##### Preliminary Effect

*Attitude toward end-of-life care*. Staff’s attitude toward end-of-life care for older adults was measured using an end-of-life care nursing attitude scale for Japanese geriatric nurses (ELNAS-JG), which measures attitudes toward end-of-life care for older adults in any end-of-life setting [40]. The ELNAS-JG consists of 26 items rated on a 5-point Likert scale, from “1 = strongly disagree” to “5 = strongly agree”; higher scores indicate more positive attitudes toward end-of-life care. The validity and reliability (Cronbach’s α = 0.96) of the ELNAS-JG were confirmed in a previous study [40]. Reliability was also confirmed using data from the present study (e.g., α ranged from 0.95 to 0.97 for various time points).

The staff’s attitude toward providing care for dying people was also examined using the Frommelt Attitudes Toward Care of the Dying Scale, Japanese Version (FATCOD-B-J) [41]. FATCOD-B-J consists of 30 items rated on a 5-point Likert scale, from “1 = strongly disagree” to “5 = strongly agree”; higher scores indicate more positive attitudes toward caring for dying people. The validity and the reliability of the FATCOD-B-J were confirmed in a previous study (Cronbach’s α = 0.85.) [41]. Reliability was also confirmed using data from the present study (e.g., α ranged from 0.76 to 0.91 for various time points).

*Interdisciplinary collaboration*. An Assessment Scale of Health Care Professionals’ Recognition of a Successful Interdisciplinary Team Approach in Health Care Facilities for the Elderly (abbreviated ITA) [42] was used. The ITA consists of 32 items rated on a 4-point Likert scale, from “0 = strongly disagree” to “3 = strongly agree”; higher scores indicate stronger perceived interdisciplinary collaboration in the respondents’ team. The validity and the reliability (Cronbach’s α > 0.9) of the ITA were confirmed in a previous study [42]. Reliability was also confirmed using data from the present study (e.g., α ranged from 0.96–0.97 for various time points).

##### Acceptability

We developed items to measure participants’ reactions to the intervention in terms of its acceptability [35], with reference to previous research [28]. Participants answered items on a 4-point Likert-type scale, rating from “1 = strongly disagree” to “4 = strongly agree.” The four items included the following: helpfulness for nurses and care workers, change in the frequency of symptom assessment, the usability of the structured documents, and improvement of overall care by the EOL Care Tool.

#### 2.5.3. Qualitative Data

After the intervention, five semi-structured focus group interviews were conducted for nine nurses and 13 care workers. We asked the participants about what was good or helpful, and what was burdensome or difficult in order to explore the reaction to the intervention (the acceptability). The interviews were digitally recorded and transcribed.

### 2.6. Data Analysis

#### 2.6.1. Quantitative Data

The analysis was conducted on participants who completed the ELNAS-JGs at T_0_ and T_3_. Descriptive statistics were calculated for participants’ characteristics. Scores on each scale were compared between T_0_ and each of the other three time points using paired t-tests (FATCOD-B-J, ELNAS-JG, ITA). We calculated adjusted *p*-values using the Holm method to correct for Type 1 error rate inflation [43]. IBM Corp. Released 2019. IBM SPSS Statistics for Windows, Version 26.0, Armonk, NY, USA, IBM Corp was used for the analysis.

#### 2.6.2. Qualitative Data

An inductive content analysis, including the process of open coding, creation of categories, and abstraction [44], was performed. The interview transcripts were read repeatedly to understand their content. Data were coded for each meaning unit. The codes were grouped into subcategories focusing on the similarities and differences in the meaning of the code. Subcategories were grouped based on the content into categories, and categories were abstracted. Categories related to positive and negative reactions to the EOL Care Tool were identified. After identifying the subcategories and categories, the codes included in each category were counted.

To ensure trustworthiness, the first author discussed the coding with authors who are experts in qualitative research, gained the opinion of an experienced researcher of end-of-life care in LTC facilities, and compared the data and findings repeatedly until reaching consensus. To ensure rigor, memos were used to record the process of analyses and the content of the discussions at seminars and meetings.

### 2.7. Ethical Considerations

First, we obtained approval to conduct the research from the director of the LTC facility. In the quantitative study, the participants were provided written and oral information regarding the study, including voluntary participation, the right to refuse participation, and confidentiality, and were ensured anonymity. We interpreted returning the questionnaire as consent to participate. In the qualitative study, the study information was provided to the participants verbally and in writing. Each participant provided written informed consent to participate. This study was approved by the institutional review board of the first author’s university.

## 3. Results

### 3.1. Quantitative Results

In all, 79 staff members (24 nurses and 55 care workers) received all 6 lectures. The number of staff members (nurses and care workers) surveyed at T_0_, T_1_, T_2_, and T_3_, was 79 (24 and 55), 79 (24 and 55), 75 (23 and 52), and 72 (22 and 50), respectively. For time points T_2_ and T_3_, the number of people surveyed decreased due to department transfers and leave of absence. Response rates at T_0_, T_1_, T_2_, and T_3_ were 87.3% (nurses, care workers: 87.5%, 87.2%), 91.1% (91.6%, 90.9%), 82.7% (86.9%, 78.8%), and 80.6% (81.8%, 78.0%), respectively. Fourteen nurses (58.3%) and 33 care workers (60%) completely answered the ELNAS-JG at both T_0_ and T_3_ and comprised the sample for the quantitative analysis.

### 3.2. Characteristics

The characteristics of the participants in the quantitative analysis are shown in Table 2. Most were female (100% of nurses, 84.4% of care workers). Many were more than 40 years old (92.8% of nurses, 63.6% of care workers).

### 3.3. Preliminary Effect

**Figure A1:**
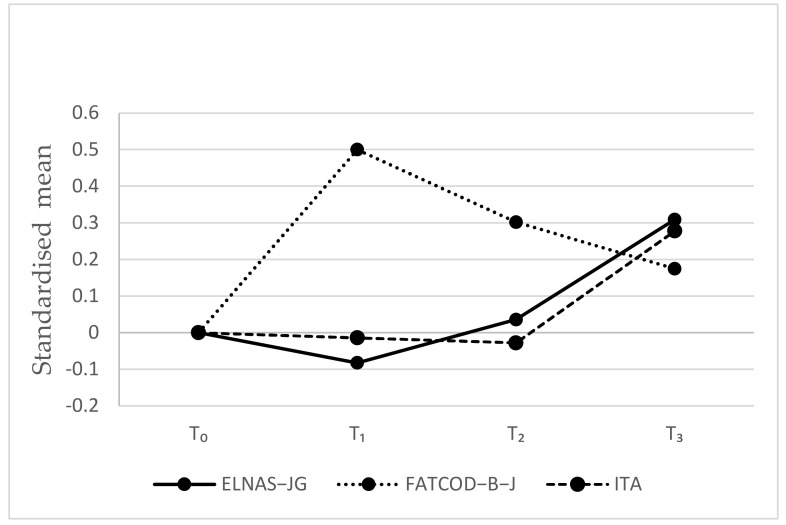
Changes in the standardized mean scores on the ELNAS-JG, FATCOD-B-J, and ITA for nurses. (*n* = 14). Note: ELNAS-JG: End-of-Life Care Nursing Attitude Scale for Japanese Geriatric Nurses; FATCOD-B-J: Japanese version of the Frommelt Attitudes Toward Care of the Dying Scale; ITA (Abbreviated): Assessment Scale of Health Care Professionals’ Recognition of a Successful Interdisciplinary Team Approach in Health Care Facilities for the Elderly. Scores at the three timepoints (T_1_, T_2_, T_3_) were standardized using the mean and standard deviation at baseline (T_0_). Z = (x − μ_0_)/σ_0_; μ_0_ and σ_0_ indicate the mean and standard deviation, respectively, at T_0_.

**Figure A2:**
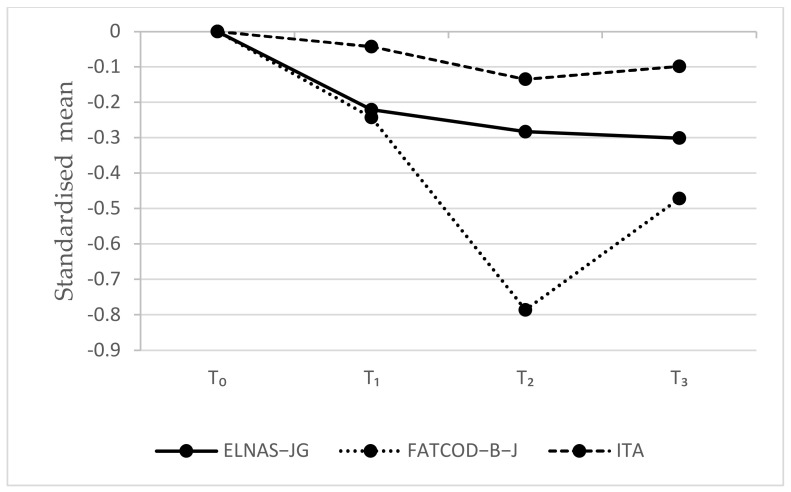
Changes in the standardized mean scores on the ELNAS-JG, FATCOD-B-J, and ITA for care workers. (*n* = 33). Note: ELNAS-JG: End-of-Life Care Nursing Attitude Scale for Japanese Geriatric Nurses; FATCOD-B-J: Japanese version of the Frommelt Attitudes Toward Care of the Dying Scale; ITA (Abbreviated): Assessment Scale of Health Care Professionals’ Recognition of a Successful Interdisciplinary Team Approach in Health Care Facilities for the Elderly. Scores at the three timepoints (T_1_, T_2_, T_3_) were standardized using the mean and standard deviation at baseline (T_0_). Z = (x − μ_0_)/σ_0_; μ_0_ and σ_0_ indicate the mean and standard deviation, respectively, at T_0_.

Table 3 shows the scores of each instrument at each time point and the results of the paired *t*-tests between baseline and the other three time points. For nurses, pairwise comparisons were significant between T_0_ and T_3_ for the ELNAS-JG and ITA (*p* < 0.05), indicating that nurses’ attitudes toward end-of-life care and interdisciplinary collaboration improved nine months after launching the intervention. In contrast, for care workers, pairwise comparison was significant between T_0_ and T_2_ in FATCOD-B-J (*p* < 0.01), indicating that care workers’ positive attitude toward care for the dying declined at three months after the launch. Appendix A (Figure A1 and Figure A2) shows nurses’ and care workers’ changes in standardized mean scores on the ELNAS-JG, FATCOD-B-J, and ITA.

### 3.4. Acceptability

Table 4 shows their evaluation regarding the acceptability of the EOL Care Tool. Over 90% of nurses and over 70% of care workers responded that the EOL Care Tool was helpful for understanding end-of-life care and that the frequency of symptom assessment increased. Over 90% of nurses and over 60% of care workers felt an improvement in overall care after intervention. In contrast, the structured documents were not perceived as user-friendly by approximately half of the nurses and care workers.

### 3.5. Qualitative Results

Analysis of focus group interviews yielded eight categories of positive and negative reactions, broadly indicating the acceptability of the EOL Care Tool from nurses’ and care workers’ perceptions. In Table 5, the ratios of nurses and care workers who made statements regarding each subcategory are shown. Participants’ quoted perceptions are presented both in the body (in *italics*) and the Appendix A (Table A1).

***Improvement in commitment to and practices for end-of-life care***. This includes improving awareness and commitment and improving the practice of end-of-life care.

*“We should provide care in consideration of what we can do for residents. I feel that such awareness among staff is probably increasing [by the tool].”*

(Nurse C, clinic)

***Improvement in interdisciplinary collaboration.*** This includes consolidation of information, sharing information, having the same point of view about resident care, and increased communication.

*“By looking at one resident’s [structured] document [including previous information from when the resident was more independent], I was able to know what the family had thought at that time.”*

(Nurse D, clinic)

***Unity between staff, residents, and their families with a common goal for better end-of-life care.*** Nurses and care workers increased communication and closeness with residents and their families with a common goal for better end-of-life care. Specifically, they engaged in more personal conversations with residents and their families and tried to reflect residents’ wishes and values in end-of-life care.

***Inspiring residents and their families to be interested in end-of-life care.*** This included catalyzing conversation about residents’ end-of-life and elevating interest in their end-of-life care.

***Preventing omission of end-of-life care for nurses and care workers.*** Preventing mistakes and ensuring consistent care among staff was mentioned by nurses in particular.

***Reluctance to address the work regarding end-of-life care.*** Participants were reluctant to provide and perceived difficulty in providing end-of-life care. This was discussed by more care workers than nurses. Some care workers felt that communication related to death and dying was not their job and felt concerned about worsening relationships between staff and residents/families. Some experienced difficulty addressing medical matters regarding end-of-life care.

*“It is hard for care workers like me to ask [residents and families] such [sensitive] things (talking about end-of-life). I’m not in a managerial position; I just write [what I hear] on [the structured document].”*

(Care worker F, high-support section)

***Psychological burden of residents and their families toward end-of-life care communication.*** Participants pointed out that residents and their families might have felt resistance and psychological burden related to the conversation regarding end-of-life. Some staff was concerned about the possibility that residents or families felt that the conversation about end-of-life care forced them to face the death of the resident.

***Increased workload.*** Participants reported increasing workloads and felt the structured document and regular conferences were troublesome.

## 4. Discussion

This study developed the EOL Care Tool, a multi-component intervention that consisted of lectures, structured documents, regular conferences, and educational support. We explored the preliminary effect and the acceptability of the EOL Care Tool for nurses and care workers. Preliminary effects were identified especially in nurses, whose attitudes toward end-of-life care and interdisciplinary collaboration were significantly improved. On the other hand, a strong preliminary effect for care workers was not identified; no significant improvement of attitudes or interdisciplinary collaboration was shown. These results suggest that this intervention currently works especially for the nurses’ attitude and interdisciplinary collaboration. Regarding acceptability, many nurses and care workers evaluated this tool positively, except for the need to modify the structured documents. In the qualitative data, nurses and care workers in general positively perceived the EOL Care Tool, although care workers showed reluctance toward end-of-life care. Therefore, acceptability was moderately shown for both nurses and care workers.

### 4.1. Preliminary Effect

Regarding the preliminary effect of this tool, score change in ELNAS-JG for nurses showed improvement in attitudes toward end-of-life care for older adults. In addition, qualitative results indicated that nurses had felt that the tool enhanced their awareness of end-of-life care in practice. Other multicomponent intervention studies using education or training had also reported improvement of staff attitude toward end-of-life care [21,23,28]. As previous studies have suggested that education regarding end-of-life care improves attitudes towards it [45,46], end-of-life care education, which is one of the components of this tool, could have contributed to the improvement of nurses’ attitudes in this study.

For care workers, preliminary effects of the EOL Care Tool were not shown. There was no significant score change in ELNAS-JG. In addition, the score decreases in FATCOD-B-J indicated a tentative negative influence on care workers’ attitudes toward caring for dying people three months after the launch of using the structured documents in clinical practice. Previous research has revealed that FATCOD-B-J scores and fear of death are negatively associated [47]: those with lower attitudes toward end-of-life care are more likely to feel fear of death. It could be considered that care workers felt and feared death more closely temporarily due to more careful observation of physical and mental symptoms of residents who were in end-of-life with this intervention. Educational support for them in clinical practice is necessary to reduce their psychological burden.

A preliminary effect was shown in interdisciplinary collaboration for nurses, but not for care workers. Nurses’ score changes in the ITA quantitatively indicated improvement in interdisciplinary collaboration. The qualitative results also indicated that improvement of interdisciplinary collaboration through the EOL Care Tool was perceived among nurses. As education differs between healthcare professionals and care workers, in terms of medical expertise and ethos/views of care, it could become a barrier to interdisciplinary collaboration between them. Here, the structured documents reflecting the views/opinions of both nurses and care workers may have facilitated interdisciplinary collaboration between nurses and care workers; in addition, the use of structured documents at regular conferences may have promoted discussion about end-of-life care for residents between nurses and care workers. The documents and conferences implemented with the EOL Care Tool could help improve interdisciplinary collaboration between nurses and care workers in LTC settings.

### 4.2. Acceptability

Potential evidence of acceptability was indicated by nurses’ and care workers’ positive evaluations of the EOL Care Tool: many nurses and care workers perceived it as promoting the understanding of end-of-life care, improving the frequency of practice (symptom management) of end-of-life care, and improving overall end-of-life care. However, the requirement to improve the EOL Care Tool was also shown, as more than half of them felt it was not easy to use the structured documents.

Qualitative results indicated acceptability of the EOL Care Tool in nurses, reporting that they perceived positively that the EOL Care Tool “prevents omission of end-of-life care for nurses and care workers”. A previous study that combined structured documents and education for end-of-life care in LTC facilities had also reported intervention brought consistency of care among staff [48]. Showing nurses and care workers what they need to do in end-of-life care may result in unified care and prevent omissions in care. The “daily records” of the structured document included items for the careful observation of residents in the dying phase and items for symptom management and required care in the dying phase: daily support, information on treatment, and caring for family. The “daily records” could have prevented participants from overlooking the necessary care for residents close to death.

In addition, categories with similar content to those in previous research on complex intervention for end-of-life care in LTCFs were found, such as improvement of attitude toward end-of-life care and improvement of interdisciplinary collaboration [23,28,49]. Meanwhile, new points of interest included “unity between staff, residents, and their families with a common goal for better end-of-life care”, which was another important qualitative finding of our research, underpinning the acceptability. This category indicates that the EOL Care Tool could help nurses and care workers, together with residents and their families, with a common goal: engaging to realize an end-of-life that reflects preferences and values. As a result of repeated conversations about end-of-life with residents and their families, closeness and relationships could have deepened between participants, residents, and their families. Because end-of-life care is defined as helping “persons who are facing imminent or distant death to have the best quality of life possible till the end of their life regardless of their medical diagnosis, health conditions, or age” [50], this tool could be expected to achieve the ultimate goal of better end-of-life care.

Acceptability for care workers was shown in the quantitative results and was also supported by qualitative data indicating positive perceptions of the EOL Care Tool. However, qualitative data simultaneously showed negative reactions by care workers toward this intervention. Therefore, acceptability for care workers seems to be moderate. Qualitative data indicated that care workers had a positive perception of the tool with respect to improving staff’s end-of-life care practices (e.g., care that reflects the wishes and preferences of residents and their families), consolidating information using the structured documents, and promoting information sharing among the staff.

On the other hand, some care workers felt reluctant to talk about end-of-life with residents and their families: the qualitative data showed that they were “reluctant to address the work regarding end-of-life care”.

Increased knowledge of and involvement in end-of-life care through this intervention might lead to a burden for care workers, who have less expertise regarding medical/palliative care than nurses [51]. Secondly, talking about end-of-life with persons for whom death is not so imminent should be difficult, especially in Japanese culture, where talking about death tends to be avoided [34]. In this study, unlike nurses, care workers may have had difficulty in talking with residents about end-of-life, as they were more used to communicating with residents who were healthy. Thirdly, some care workers felt that end-of-life care conversations with relatively healthy residents were outside their scope of practice. Such conversations might have been perceived as beyond their original/conventional tasks and also as difficult and extra work.

For enhancing the acceptability of the EOL Care Tool to care workers, revision to reduce the burden of end-of-life care conversations should be examined hereafter. As care workers need hands-on education more than didactic education [52], they may have experienced difficulty in linking their learning from the education of this tool to their care practice. More appropriate education that can be reflected in their practice is necessary.

### 4.3. Implications for Further Research

Whereas many previous studies regarding multicomponent intervention for end-of-life care in LTCFs have analyzed and discussed nurses, care workers, and other participants together [10,16,23,24,28,48,49], analyzing nurses and care workers separately allowed our study to differentiate the influence and acceptability of this tool on each group and reveal related issues that require attention. The results of this study indicated the preliminary effect and the acceptability of this tool for nurses, although for care workers the preliminary effect was not shown and acceptability was moderate. However, some aspects of the intervention require revision. Decreasing the burden for care workers and allowing more care workers to increase the acceptability of this tool could increase its feasibility. Therefore, the required revisions include changes for ease of use of the structured documents; practical education such as nurse shadowing in care provision; a support system for care workers in end-of-life care; psychological support for care workers, especially regarding conversations in end-of-life care practice; and education about end-of-life care conversations and psychological support for residents and families. Further comparative research is needed to confirm the feasibility and effects of a revised EOL Care Tool.

Additionally, our study does not measure the outcomes for residents and their families using this tool. One study of an end-of-life care program conducted in geriatric wards in Belgium found that evaluations by families contrasted with those by nurses [53]. It is necessary for future studies to examine the feasibility for residents and families carefully because end-of-life care involves extremely sensitive topics.

### 4.4. Limitations

This study has several limitations. First, participants in this study were from only one LTC facility, with a small sample size, which might limit generalizability to other LTC facilities. It will be necessary to interpret the preliminary effect and acceptability of this intervention carefully. Given that this facility employed a CNS, the quality of end-of-life care might be higher than in other LTC facilities in Japan, as CNSs in LTC settings are quite rare in Japan. Additionally, further research with larger sample sizes should be needed to improve the intervention and examine its efficacy.

Second, ELNAS-JG was developed for nurses to measure their attitudes toward end-of-life care for older adults in any end-of-life setting, though we confirmed the face validity of the survey items with both nurses and care workers. This might affect the result of this study.

Third, external validity could be compromised by the characteristics of the participants and the interventions in other sites and/or countries, because the thought to the end of life, the culture, and staffing in the LTC setting is different in each country.

## 5. Conclusions

In this study, we explored the preliminary effect and acceptability of this end-of-life tool. This study showed the preliminary effect and acceptability for nurses and revealed the limited acceptability among care workers. It is thus necessary to revise the tool to need modification and examine the effect of the revised tool with a more robust research design. Further research should include residents and their families as participants to measure clients’ outcomes and examine the feasibility of the EOL Care Tool for them, as it was confirmed that the EOL Care Tool was potentially feasible for both nurses and care workers to some extent.

## Figures and Tables

**Table 1:** Components of the EOL Care Tool.

	Components	Implementation
**(1)** End-of-life care lectures	The lectures comprised the following six modules: (Each lecture lasted approximately 60 min.) “What is end-of-life care?” “Cultural and ethical issues” “Communication and loss, grief, and bereavement” “Pain management” “Symptom management” “Care in dying phase and death” “How to use structured documents?”	Lectures for staff were conducted as follows: Researchers provided lectures for staff. Each module was conducted several times, in face-to-face lectures and, DVD-recorded lectures, to facilitate the participation of all staff (nurses and care workers).
**(2)** Structured documents shared by the interdisciplinary team	The document consists of four types of sheets: *“Assessment sheet” (initial, ongoing)* These two documents aimed to confirm and share the thoughts and wishes of residents and their families about their life and end-of-life between staff. The documents included the following information: (a) residents’ wishes and anxieties about end-of-life (b) family members’ wishes and anxieties about end-of-life care (c) residents’ level of cognitive function and activities of daily living, current medical treatment, and interest in treatment related to end-of-life care (d) family members’ interest in disease course and treatment related to end-of-life care *“Conference sheets” (1, 2)* These documents were used to assess the residents’ physical/psychological condition and care process, and to discuss care for expected condition changes. They contained the following information to assess changes/decline in residents’ physical/psychological conditions: weight, activity, walking, depression, food intake, etc., based on existing literature on frailty. These sheets comprised three sections: discussion of physical and mental health assessment, information shared with family members, and physician’s judgment (if any). Shared information was important to promote shared decision-making with family members and to avoid conflict among staff and family members due to recognition gaps. *“Daily records”* This document aimed to standardize the assessment and care provided by nurses and care workers. It contained items for symptom management and a list of required care practices during the dying phase. Required care practices included daily support, information on treatment, and caring for family. This document was a sheet typically used for residents who were judged as being close to death in the regular conference. *“Preparation sheet for imminent death”* This document aimed to ensure that the required activities and rituals during the period of dying and after death are performed properly. It contained items such as procedures at dying and death and bereavement care.	The structured document was implemented as follows: Staff (nurses and care workers) started using structured documents for residents’ care. Both nurses and care workers used the structured documents, in parallel with existing records. Staff talked with residents and their families about the resident’s end-of-life care and recorded the information in the structured documents before the regular conference.
**(3)** Regular conferences on end-of-life care	Regular conferences were conducted by nurses, care workers, and sometimes clerks in the long-term-care facility. In the conferences, discussions were to be conducted regarding the management of changes in residents’ wishes/anxieties and decline/recovery in their physical/psychological condition. The condition of their frailty or dying trajectory and the prospects of their health condition were to be discussed.	Implementation of the regular conferences was as follows: Staff conducted regular conferences for end-of-life care of residents, using the assessment sheet and conference sheet. The interval of discussion was decided by staff according to residents’ physical condition, in consultation with nurse/care worker managers and doctors: one week, one month, three months, or six months (considering the typical workflow in Japanese long-term care facilities). Usually, the interval was adjusted reversibly and shortened with resident deterioration, but it could also be extended if their condition improved.
**(4)** Educational support	To set up a support system for the implementation of the EOL Care Tool, researchers provided the managers of nurses and care workers an educator role and asked them to adjust the conference schedule and support the staff with clinical implementation. Educators supported staff using a manual created to implement the intervention (the manual included explanations of how to use this tool). Their roles included advising on how to gather and assess residents’ information using this tool, facilitating communication about end-of-life with staff and residents/their families, encouraging staff through face-to-face conversation and regular conferences, and helping the staff cope with anxiety.	Implementation of educational support was as follows: The nurse managers and care worker managers, as educators, supported the staff in clinical cases and in the use of structured documents through direct guidance and discussions in conferences and practice.

**Table 2:** Demographic data (*n* = 47).

		Nurses (*n* = 14)	Care Workers (*n* = 33)
		*n* (%)	*n* (%)
Gender	Female	14 (100.0)	28 (84.8)
	Male	0 (0.0)	5 (15.1)
Age (years)	≤29	0 (0.0)	9 (27.3)
	30–39	1 (7.1)	3 (9.1)
	40–49	4 (28.6)	12 (36.4)
	50–59	8 (57.1)	8 (24.2)
	≥60	1 (7.1)	1 (3.0)
Occupation (multiple answers allowed)	RN	13	0
	LPN	1	0
	Certified care worker	0	27
	Social worker	0	2
	Licensed home-helper	0	9
	Care manager	0	4
	Other	0	1
Educational level	High school	0 (0.0)	6 (18.1)
	Vocational school	12 (85.7)	11 (33.3)
	Junior college	1 (7.1)	7 (21.2)
	University	1 (7.1)	9 (27.3)
Years of experience in their profession	≤5	0 (0.0)	10 (30.3)
	6–10	0 (0.0)	11 (33.3)
	11–15	3 (21.4)	6 (18.2)
	≥16	11 (78.5)	6 (18.2)
Years working in the current workplace	≤5	8 (57.1)	21 (63.6)
	6–10	4 (28.6)	6 (18.1)
	11–15	0 (0.0)	4 (12.1)
	≥16	2 (14.3)	0 (0.0)
	Missing	0 (0.0)	2 (6.1)

RN, registered nurse; LPN, licensed practical nurse.

**Table 3:** Changes in nurses’ and care workers’ attitudes toward end-of-life care and interdisciplinary collaboration with the EOL Care Tool (*n* = 47).

Measure	T_0_		T_1_		T_2_		T_3_		Adjusted *p*-Value		
	*n*	Mean (SD)	*n*	Mean (SD)	*n*	Mean (SD)	*n*	Mean (SD)	T_0_-T_1_	T_0_-T_2_	T_0_-T_3_
**Nurses (*n* = 14)**											
ELNAS-JG	14	82.4 (19.4)	13	80.8 (17.0)	14	83.1 (20.4)	14	88.4 (20.5)	0.99	0.74	0.02 *
FATCOD-B-J	13	115.5 (12.6)	12	121.8 (13.9)	13	119.3 (14.0)	14	117.7 (14.6)	0.09	0.21	0.11
ITA	12	56.0 (14.4)	13	55.8 (12.3)	12	55.6 (13.9)	14	60.0 (13.4)	0.99	0.93	0.02 *
**Care workers (*n* = 33)**											
ELNAS-JG	33	77.2 (11.3)	30	74.7 (11.4)	28	74.0 (15.0)	33	73.8 (15.2)	0.25	0.22	0.27
FATCOD-B-J	31	114.5 (7.0)	29	112.8 (7.6)	29	109.0 (8.7)	31	111.2 (9.2)	0.11	0.00 **	0.07
ITA	31	58.8 (16.3)	31	58.1 (15.1)	30	56.6 (15.0)	33	57.2 (14.9)	0.73	0.99	0.99

Note: Participants who completed the ELNAS-JG at both T_0_ and T_3_ were included in the analysis. Missing data were excluded for each test. Missing data were treated using pairwise deletion. SD, standard deviation; ELNAS-JG: End-of-Life Care Nursing Attitude Scale for Japanese Geriatric Nurses; FATCOD-B-J: Japanese version of the Frommelt Attitudes Toward Care of the Dying Scale; ITA (Abbreviated): Assessment Scale of Health Care Professionals’ Recognition of a Successful Interdisciplinary Team Approach in Health Care Facilities for the Elderly. Level α = 0.05. * *p* < 0.05. ** *p* < 0.01.

**Table 4:** Acceptability of the EOL Care Tool for nurses and care workers (*n* = 47).

	Nurse (*n* = 14)				Care Worker (*n* = 33)			
	*n* (%)				*n* (%)			
	Strongly Agree	Agree	Disagree	Strongly Disagree	Strongly Agree	Agree	Disagree	Strongly Disagree
The frequency of symptom assessment for residents who are dying has increased since the introduction of the EOL Care Tool.	7 (50.0)	7 (50.0)	0 (0.0)	0 (0.0)	11 (33.3)	14 (42.4)	8 (24.2)	0 (0.0)
The EOL Care Tool has helped me understand appropriate care for residents who are dying.	5 (35.7)	8 (57.1)	1 (7.1)	0 (0.0)	5 (15.2)	21 (63.6)	6 (18.2)	1 (3.0)
The overall care for those who are dying has improved since the implementation of the EOL Care Tool.	2 (14.3)	11 (78.6)	1 (7.1)	0 (0.0)	5 (15.2)	16 (48.5)	11 (33.3)	1 (3.0)
The EOL Care Tool document is easy to use for staff.	1 (7.1)	5 (35.7)	7 (50.0)	1 (7.1)	1 (3.0)	12 (36.4)	17 (51.5)	3 (9.1)

**Table 5:** Nurses’ and care workers’ perceptions of the EOL Care Tool (*n* = 22).

Category	Subcategory	Nurse (*n* = 9)	Care Worker (*n* = 13)
Improvement in commitment to and practices for end-of-life care	Improving staff’s awareness of and commitment to end-of-life care	77%	46%
	Improving staff’s daily-care practices (e.g., symptom management, supporting daily life, looking back at residents’ care goals)	33%	38%
	Improving staff’s end-of-life care practices (e.g., providing care that reflects the wishes and preferences of residents and their family members)	66%	76%
	Learning end-of-life care and improving skills	22%	38%
Improvement in interdisciplinary collaboration	Consolidating information about residents’ and family members’ wishes, care, and conditions	44%	53%
	Sharing information about residents’ and family members’ wishes, care, and conditions through documents and conferences	55%	46%
	Conducting frequent conferences where nurses and care workers can have discussions from the same perspective regarding residents’ care	44%	30%
	Increasing communication among nurses and care workers in care practice	33%	7%
	Cooperating well within the section	44%	23%
Unity between staff, residents, and their families with a common goal for better end-of-life care	Increasing communication and closeness among staff, residents, and their families	55%	23%
	Having more personal conversations regarding end-of-life care with residents and their families	22%	7%
	Uniting staff, residents, and their families for end-of-life care	55%	7%
Inspiring residents and their families to be interested in end-of-life care	Providing opportunities to discuss end-of-life care	11%	46%
	Improving residents’ and families’ interest in residents’ end-of-life	11%	23%
Preventing omission of end-of-life care for nurses and care workers	Preventing oversights in care practice and symptom observation	33%	7%
	Unifying care practices among staff	22%	7%
Reluctance to address the work regarding end-of-life care	Belief that having conversations related to dying is not the job of care workers	0%	15%
	Resistance to end-of-life care conversations that might worsen relationships with residents and their families or create a psychological burden for them	11%	46%
	Facing difficulties and lacking confidence in addressing medical matters regarding end-of-life care	11%	38%
	Barriers to talking about end-of-life care in ways other than face-to-face conversations with family members	22%	38%
Psychological burden of residents and their families toward end-of-life care communication	Residents and family members are resistant to end-of-life care communication	0%	53%
	Residents and family members sense a psychological burden related to end-of-life care communication	11%	15%
Increased workload	Increased workload owing to the documentation and conferences	22%	15%
	Finding it troublesome to create extra records and/or organize conferences	33%	23%

Note: The numbers in the right column indicate the percentages of nurses and care workers, respectively, who made statements about each subcategory.

## Data Availability

The data are not publicly available, as they contain information that could compromise our participants’ anonymity.

## UMIN Clinical Trial Registry

UMIN000022579.

## Appendix A

**Table A1:** Categories and interview excerpts: Nurses’ and care workers’ perceptions of the EOL Care Tool.

Category	Subcategory	Interview Excerpts
Improvement in commitment to and practices for end-of-life care	Improving staff’s awareness of and commitment to end-of-life care	*“I think [that the] awareness among staff [toward end-of-life care for residents] was less before. I think that as a resident approaches death, we should give him or her more comfort. Or we should provide care taking into consideration what we can do for the residents. I feel that such awareness among staff is probably increasing.”* (Nurse C, clinic)
	Improving staff’s daily-care practices (e.g., symptom management, supporting daily life, looking back at residents’ care goals)	*“In the care [for residents] at the end of life, for example, when the rubber sheets [water-repellent sheets] were wrinkled…because the wrinkles might make them feel uncomfortable, I was careful [not to wrinkle the sheets]. In that way, I thought about caring for residents.”* (Care worker G, high-support section)
	Improving staff’s end-of-life care practices (e.g., providing care that reflects the wishes and preferences of residents and their family members)	*“I think we were able to do what we could until the end, in consideration of the things valued by residents and their families.”* (Care worker C, high-support section)
	Learning end-of-life care and improving skills	*“How to care for residents who are approaching death… I felt I had improved the level [of care]. I also believe I learned things.”* (Care worker G, high-support section)
Improvement in interdisciplinary collaboration	Consolidating information about residents’ and family members’ wishes, care, and conditions	*“There were residents…who were approaching death when this [tool implementation] began. Now, everyone can see what kind of care we should provide for such residents. The staff can see the direction of care just by looking at the documents.”* (Care worker E, high-support section)
	Sharing information about residents’ and family members’ wishes, care, and conditions through documents and conferences	*“By looking at a resident’s document [structured document including information from when the resident was more independent], I was able to know what the family had thought at that time. I was able to know what I had not previously known [about the family’s attitudes].”* (Nurse D, clinic)
	Conducting frequent conferences where nurses and care workers can have discussions from the same perspective regarding residents’ care	*“About sharing [information] between nurses and care workers…it’s great to be able to discuss things together while sharing documents.”* (Care worker E, high-support section)
	Increasing communication among nurses and care workers in care practice	*“When I couldn’t ask a resident for information but had to gather it…I developed relationships with staff from other sections, as well as families…and had conversations with them.”* (Nurse G, clinic)
	Cooperating well within the section	*“The negative side was that we couldn’t really share information [with another section]. [But] in the clinic, we can share [information].”* (Nurse C, clinic)
Unity between staff, residents, and their families with a common goal for better end-of-life care.	Increasing communication and closeness among staff, residents, and their families	*“Staff can enhance their closeness with the residents. In that sense, I thought it [the EOL Care Tool] was good.”* (Nurse G, clinic)
	Having more personal conversations regarding end-of-life care with residents and their families	*“I thought it was good for me to be able to talk deeply with residents’ families.”* (Nurse F, high-support section)
	Uniting staff, residents, and their families for end-of-life care	*“By having common goals, we were able to join forces to provide end-of-life care for residents using this [the structured document].”* (Nurse E, clinic)
Inspiring residents and their families to be interested in end-of-life care	Providing opportunities to discuss end-of-life care	*“I think it [the tool] provided an opportunity for families to think [about end-of-life wishes and so on].”* (Care worker J, low-support section)
	Improving residents’ and families’ interest in residents’ end-of-life	*“One family member said, ‘I think I have to talk things over with my brothers. Could you provide us with relevant materials if there [are any]?’”* (Care worker J, low-support section)
Preventing omission of end-of-life care for nurses and care workers.	Preventing oversight in care practice and symptom observation	*“The checklist items help us to avoid missing anything.”* (Nurse G, clinic)
	Unifying care practices among staff	*“What we have to do now is unification, I think. Perhaps [we will proceed] by doing what is written in the [structured] documents.”* (Nurse B, clinic)
Reluctance to address the work regarding end-of-life care	Belief that having conversations related to dying is not the job of care workers	*“It is hard for care workers like me to ask [residents and families] such [sensitive] things (talking about end-of-life). I’m not in a managerial position; I just write [what I hear] on [the structured document].”* (Care worker F, high-support section)
	Resistance to end-of-life care conversations that might worsen relationships with residents and their families or create a psychological burden for them	*“We called the resident’s family and asked about their hopes for the resident’s end-of-life. But I really didn’t want to do it at first. We already had a relationship [with the resident and his/her family], and the resident was well [in good health]. So, why do I have to talk with them about end-of-life? I thought [about] it. I felt very resistant [at that time].”* (Care worker D, high-support section)
	Facing difficulties and lacking confidence in addressing medical matters regarding end-of-life care	*“Care workers will also communicate [medical information to residents or families], but I think it is difficult [for care workers].”* (Care worker E, high-support section)
	Barriers to talking about end-of-life care in ways other than face-to-face conversations with family members	*“I thought it was painful and difficult for staff to talk to families about such serious topics over the phone.”* (Care worker F, high-support section)
Psychological burden of residents and their families toward end-of-life care communication	Residents and family members are resistant to end-of-life care communication	*“The topic of life and death is a sensitive issue, isn’t it? Some residents who said they were well [in good health] wondered why they now had to talk about such things [end-of-life topics].”* (Care worker M, low-support section)
	Residents and family members sense a psychological burden related to end-of-life care communication	*“When the staff talk about such things [end-of-life], the residents would have negative thoughts. One person asked, ‘Will I die soon?’”* (Care worker F, high-support section)
Increased workload	Increased workload owing to the documentation and conferences	*“Documentation must be made easier. There are too many records to create.”* (Nurse B, clinic)
	Finding it troublesome to create extra records and/or organize conferences	*“The number of conferences is gradually increasing. I found it a bit troublesome because we talk with residents and their families about the same topics repeatedly.”* (Nurse C, clinic)
